# Challenging Conventional Views: An Uncommon Case of Left-Sided Endocarditis in an IV Drug User – A Case Report

**DOI:** 10.51894/001c.144650

**Published:** 2025-09-30

**Authors:** Afeda Taher

**Affiliations:** 1 Department of Internal Medicine Detroit Medical Center-Sinai Grace Hospital

## 126

### INTRODUCTION

Intravenous drug use is a well-known risk factor for Infective endocarditis. With rising rates of substance abuse, hospitals are witnessing an increase in IE-related admissions. Although right-sided IE has long been associated with IVDU, recent trends show a

growing number of left-sided IE cases in this population.

### CASE REPORT

A 48-year-old female with PMH of HIV, CVA, and IVDU presented with AMS. BP of 98/71 mmHg, a HR of 152/min, and a Temp of 39. A CT scan identified stroke in the left medial occipital lobe, left pleural effusion, splenomegaly with a chronic subcapsular abscess, and multiloculated left perirenal abscess.TEE revealed an abscess on the anterior mitral leaflet. Cultures from blood and splenic fluid both grew MRSA. Managed by pigtail catheter drainage for the splenic, renal abscesses, and pleural effusion and received an I/V antibiotic, later transferred to a rehabilitation facility for continued care.

### DISCUSSION

A comprehensive review of 161 studies and reports involving 26 cases of active IVDU suggests that left-sided IE is an important concern in this population. In these reports, 81% involved isolated left-sided IE, while 19% involved both right- and left-side. The mitral valve was affected in 70% and the aortic valve in 58%. MRSA infection is responsible for 50% of infections. Septic emboli in 65% of patients affects the brain, lungs, spleen, and kidneys. 27% incidence of circulatory shock, hospital mortality rate of 40%. These indicate that left-sided IE in IV drug users is more prevalent and has a greater risk of severe complications due to the higher likelihood of systemic embolization.

### CONCLUSION

Left-sided infective endocarditis in individuals with a history of intravenous drug use remains an underrecognized yet highly serious condition. Both the case presented here and the existing literature underscore the importance of early diagnosis through comprehensive imaging and the implementation of aggressive, targeted antimicrobial therapy, combined with a multidisciplinary treatment strategy. Prompt, coordinated management is vital to reducing the high morbidity and mortality associated with this condition. Furthermore, the complexity of left-sided IE in IVDU patients highlights the need for additional research, its pathogenesis, and the development of optimal therapeutic approaches.

